# The Orbitoscope, a six-axis macro-imaging robot for photogrammetric 3D-digitization of insects and other small specimens

**DOI:** 10.3897/zookeys.1277.177740

**Published:** 2026-04-14

**Authors:** Toivo Aukusti Ylinampa, Urmas Kõljalg

**Affiliations:** 1 Institute of Ecology and Earth Sciences, Faculty of Science and Technology, Department of Botany, University of Tartu, J. Liivi 2, Tartu, Estonia Institute of Ecology and Earth Sciences, Faculty of Science and Technology, Department of Botany, University of Tartu Tartu Estonia; 2 Research Group for Biological Informatics, University of Tartu Natural History Museum, Tartu, Estonia Research Group for Biological Informatics, University of Tartu Natural History Museum Tartu Estonia

**Keywords:** 3D-reconstruction, digitization, entomology, focus stacking, method, structure-from-motion

## Abstract

Natural history collections contain vast numbers of small, fragile specimens whose morphology is difficult to capture using conventional 2D-imaging. Photogrammetric 3D-reconstruction from multi-view photographs can preserve surface colour and enable scaled measurement, but at macro magnification it typically requires dense viewpoint coverage with high overlap and extended depth-of-field (EDOF) imagery. Existing robotic systems often achieve viewpoint variation by rotating and tilting the specimen, which can be limiting for elongated, heavy, or fragile mounts and for objects whose geometry may change when reoriented. We present the Orbitoscope, an open-source six-axis macro-imaging robot that keeps the specimen stationary while moving the camera in translation (X–Y–Z) and orientation (A–B) around it, with a dedicated stacking axis (C) to acquire focus stacks automatically. We demonstrate the workflow by digitizing six insect specimens and generating scaled, textured 3D models suitable for preservation, measurement, and online dissemination. Basic measurement validation on one specimen showed a mean absolute percent error of 0.52% (max. 0.88%) relative to calibrated microscope reference measurements. Hardware, software, and documentation are openly released, with detailed build and operation instructions archived separately as a technical package.

## Introduction

Natural history collections contain valuable information about our environment, ecology, and insect evolution over time (Johnson and Owens 2023), and digitization of these collections has become a significant challenge (Berents et al. 2010; Vollmar et al. 2010; Moore 2011; Beaman and Cellinese 2012; Blagoderov et al. 2012; Mantle et al. 2012; Hudson et al. 2015; Martins et al. 2015; Mathys et al. 2015; Galantucci et al. 2016, 2018; Erolin et al. 2017; Mertens et al. 2017; Nguyen et al. 2017; Qian et al. 2019; Brecko and Mathys 2020). However, handling and examining fragile physical specimens makes them susceptible to damage, reducing the practical value of collections. Digital surrogates, such as 2D images, can be shared and archived in online databases, reducing handling of physical specimens. However, as insects are 3-dimensional objects, regular 2D images cannot capture all details, such as volume and surface area (Nguyen et al. 2014; Tatsuta et al. 2017; Ströbel et al. 2018). Even 1D measurements from 2D images are challenging due to parallax errors and intra-observer variability (Ströbel et al. 2018; Qian et al. 2019; Brecko and Mathys 2020). Therefore, 3D imaging is considered the desired “gold standard” (Wheeler et al. 2012).

There are multiple ways of creating 3D models of insects. Micro-CT has been used to digitize small biological specimens (Metscher 2009; Faulwetter et al. 2013), but it does not capture surface colour because it primarily measures X-ray attenuation. The same is true for scanning electron microscopy (Akkari et al. 2013; Cheung et al. 2013). Laser and structured-light scanners recover geometry and may provide colour when paired with a co-registered camera system (Aguilera and Lahoz 2006; Polo and Felicísimo 2012; Schöning and Heidemann 2016; Song et al. 2018). Also, these techniques struggle with tiny (under ~1 mm-sized) objects (Kuzminsky and Gardiner 2012; Cardaci and Versaci 2013). Photogrammetric SfM (Structure-from-Motion) is a scanning technique that retains the object’s natural colours (Ullman 1979; Seitz et al. 2006; Szeliski 2011; Westoby et al. 2012; Mathys et al. 2013; Schönberger and Frahm 2016a). It uses regular 2D images from different perspectives and generates a 3D model as output. In the first phase, the software detects and matches local image features across overlapping photographs using feature detectors/descriptors (e.g., SIFT among others; Lowe 2004). Robust estimation methods (e.g., RANSAC; Fischler and Bolles 1981) are then used to infer camera poses from these correspondences, followed by multi-view stereo (MVS) reconstruction to generate a dense 3D point cloud (Schönberger et al. 2016b). Photogrammetry can support quantitative measurements when the reconstruction is correctly scaled and the image set supports reliable pose estimation and dense surface recovery (Lauria et al. 2022). In practice, reconstruction fidelity depends on sharp, well-exposed images with minimal motion blur, sufficient depth-of-field (or extended depth-of-field, EDOF), and—critically—appropriate viewpoint selection with high overlap and comprehensive surface coverage; specimen appearance (e.g., translucency, specular highlights, low texture) and self-occlusions can further degrade reconstruction quality. Thin structures, such as antennae and hairs, remain challenging to reconstruct in 3D even with high-quality imagery (Simões et al. 2012).

Insect drawer 2D digitization with regular objectives (Mantle et al. 2012) is affordable but may not provide the resolution and depth of field needed for fine morphological detail. For improved sharpness across specimen depth, EDOF imaging is commonly used (Luhmann et al. 2013; Brecko et al. 2014). Because depth of field is shallow at macro magnifications, EDOF requires acquiring a focus stack by translating the camera (or optics) along the camera-to-specimen axis and combining the sharp regions in post-processing (Gallo et al. 2014; Clini et al. 2016; Kontogianni et al. 2017). High-quality photogrammetry typically requires dense viewpoint sampling with strong inter-image overlap and complete surface coverage, often resulting in hundreds of viewpoints depending on specimen complexity and size. When EDOF stacks are acquired at each viewpoint, the total number of captured frames can increase to many thousands, making manual acquisition impractical and motivating robotic solutions.

We are aware of several robotic systems designed for insect-scale photogrammetry. Nguyen et al. (2014) introduced a three-axis approach in which the specimen is rotated and tilted to provide viewpoint diversity while the camera translates along one axis; their workflow already includes focus stacking to extend depth of field, even if not described using the later "multi-view EDOF" terminology. Subsequent systems refined this general concept by integrating more standardized illumination, automation, and photogrammetry pipelines (e.g., Ströbel et al. 2018; Plum and Labonte 2021; Doan and Nguyen 2024). In these specimen-motion designs, viewpoints are typically sampled around a fixed aiming point near the object centre, which is effective for many pinned insects but can be limiting when (i) the region of interest is not well approximated by a single orbit centre (e.g., elongated objects or mounts with substantial depth variation), (ii) reorientation may deform soft specimens or disturb fragile mounts, or (iii) the specimen or mounting hardware is inconvenient to rotate/tilt due to mass or mechanical constraints.

Here we invert the common strategy. The Orbitoscope comprises a translational stage (X–Y–Z) that positions the camera in 3D space, a rotational stage (A–B) that controls azimuth and elevation around the specimen, and a stacking stage (C) that translates the camera along the optical axis to acquire focus stacks. While general-purpose motion-control platforms such as COPIS (COPIS 2024, https://github.com/YPM-Informatics/COPISClient) can, in principle, support focus stacking by repeating captures at multiple focus distances, the Orbitoscope integrates an explicit stacking axis and an automated sharpness-driven acquisition routine, enabling consistent per-view EDOF imaging without relying on lens refocusing. This six-axis, specimen-stationary design provides greater geometric flexibility than three-axis specimen-motion systems: it supports orbiting around different aiming points, captures only selected angles at high detail when needed, and digitizes fragile, heavy, deformable, or otherwise difficult-to-reorient specimens.

In the present study, we demonstrate the workflow on pinned insects; additional applications enabled by specimen-stationary camera motion (e.g., non-pinned mounts, fragile/deformable objects, or targeted high-resolution imaging of specific regions) are discussed as future work. Specifically, we (i) describe the six-axis specimen-stationary hardware and open-source control software, (ii) provide an end-to-end acquisition and reconstruction workflow for multi-view EDOF photogrammetry, and (iii) report basic dimensional validation against calibrated microscope measurements.

## Materials and methods

### Overview of the workflow

The Orbitoscope acquires photogrammetric image sets by moving the camera around a stationary specimen (“camera orbiting”). At each camera viewpoint, the system acquires a focus stack and then combines it into an extended depth-of-field (EDOF) image. The resulting EDOF images are then used for photogrammetric 3D reconstruction. Robot control and image acquisition were performed on an Ubuntu workstation (Ubuntu 22.04 LTS; Intel i7 8-core 5 GHz; 16 GB DDR4 RAM; NVIDIA GTX1080). Focus stacking and photogrammetric reconstruction were performed on a separate computer (Apple M1 Max; 32 GB RAM). Control software (Python and Arduino firmware, arduino.cc) is open-source and released under the MIT license. Detailed build instructions, wiring diagrams, firmware command references, and calibration guides are archived as a technical package (Orbitoscope 2025, Zenodo doi: https://doi.org/10.5281/zenodo.18563286) and mirrored on GitHub.

### Axis nomenclature and motion subsystems

**Figure 1.**
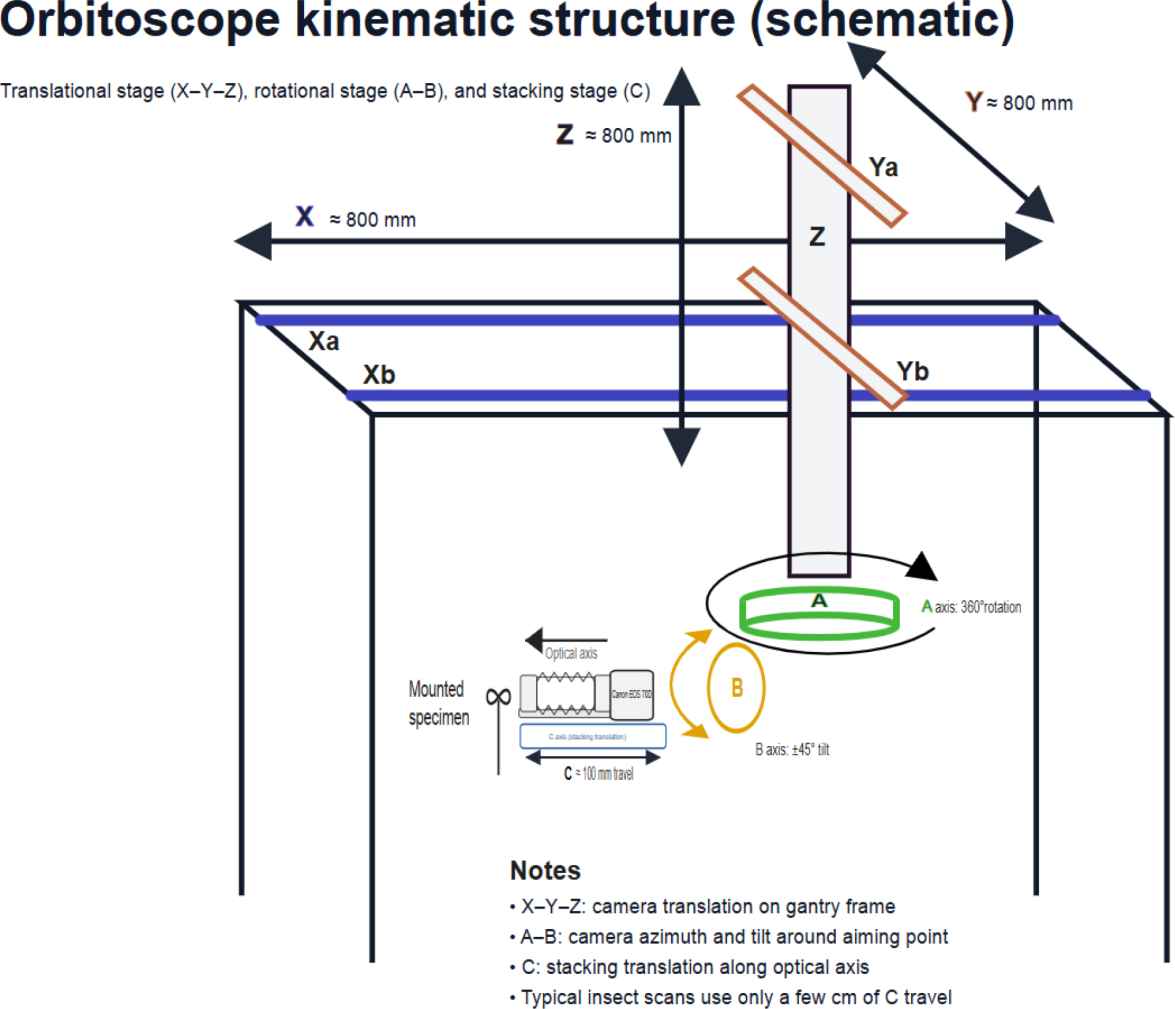
Orbitoscope motion architecture (schematic). The camera orbits around a stationary specimen using a translational stage (X–Y–Z; ~800 mm travel per axis) and a rotational stage (A, B; A = 360° azimuth rotation, B ≈ ± 45° tilt). A dedicated stacking axis (C) translates the full camera–bellows–lens assembly approximately along the optical axis for focus stacking (mechanical travel ~200 mm; ~100 mm usable in the present build).

**Figure 2.**
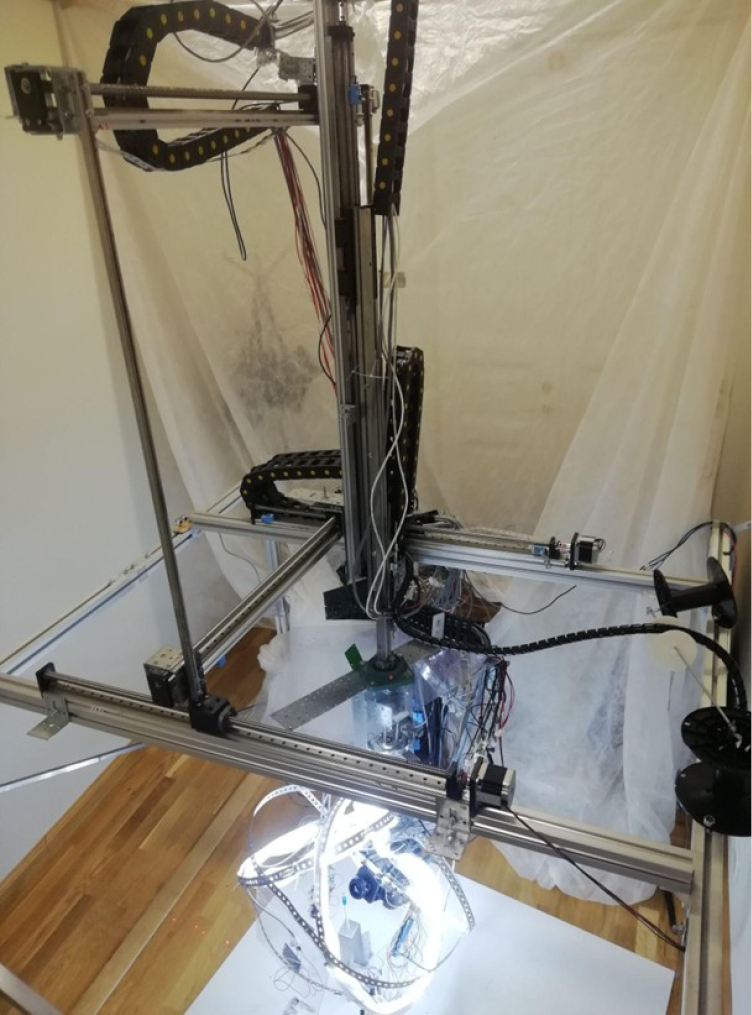
Orbitoscope prototype (overall view). Photograph of the assembled six-axis imaging robot showing the aluminium frame and the camera-motion stages: the XYZ translational gantry supporting the camera carriage, the AB rotation/tilt head, and the C-axis stacking rail carrying the camera–bellows–lens imaging module. The specimen remains stationary during acquisition while the camera moves around it to capture multi-view focus stacks for subsequent EDOF stacking and photogrammetric reconstruction.

The Orbitoscope comprises three motion subsystems: (i) a translational stage (X–Y–Z) that positions the camera in 3D space, (ii) a rotational stage (A–B) that controls camera azimuth and elevation around the specimen, and (iii) a stacking stage (C) that translates the camera approximately along the optical axis to acquire focus stacks for EDOF imaging. This convention reserves X–Y–Z for translation consistent with common 3D software coordinate frames and separates viewpoint control (A–B) from focus stepping (C). In the current build, nominal travel ranges of the translational axes are approximately 800 mm for X, Y, and Z. The A axis provides continuous 360° rotation, and the B axis provides approximately ± 45° tilt (exact range depends on mechanical clearance). The C-axis is a linear stage with ~200 mm mechanical travel; due to cabling/clearance, ~100 mm is currently usable. Routine insect scans typically require only a few centimetres of C-axis travel (Figs 1, 2).

### Hardware architecture

The system is constructed from modular aluminium profiles and linear stages supporting camera motion around a stationary specimen, with a total mass of approximately 80 kg and a footprint of approximately 1.5 m × 1.5 m. Images were acquired with a Canon EOS 70D (5472 × 3648 pixels; pixel size ≈ 4.1 µm) coupled to a PB-6 bellows and an EL-Nikkor 50 mm f/2.8 enlarging lens. During scanning, bellows extension was fixed and focus stacking was performed by translating the entire camera–bellows–lens assembly along the C-axis, rather than refocusing the lens, which keeps magnification constant within a scan.

The effective object-space sampling (µm per pixel) depends on the imaging magnification (M) and is given by: sampling = 4.1 µm / M. For example, at M = 1× the sampling is ~4.1 µm/pixel, at M = 2× ~2.05 µm/pixel, and at M = 3× ~1.37 µm/pixel. In practice, the smallest reliably recoverable features are typically several pixels wide and are further limited by residual vibration, focus-stacking artifacts, surface reflectance/translucency, and the photogrammetric reconstruction step; thus, object-space sampling should be interpreted as an upper bound on recoverable detail rather than a guaranteed spatial resolution.

Illumination is provided by a surrounding diffuse lighting structure designed to reduce hard shadows and stabilize appearance across viewpoints for photogrammetry. To facilitate consistent placement of the specimen at the imaging origin, we used an alignment aid consisting of two low-power laser pointers mounted such that their beams intersect at the desired specimen centre. During setup, the specimen is positioned so that the intersection coincides with the target point about which the camera orbit is defined. Full mechanical drawings, part numbers, suppliers, wiring diagrams, and assembly documentation are provided in the archived technical package.

### System cost

An approximate component-level cost summary for the build used in this study is provided in Table 1.

**Table 1.** The system cost. Note: An Agisoft Metashape Standard is sufficient for the reconstruction steps used here; an educational license was used in this study.

Component	Price in Euros
Frame	700
Camera, bellows, lens, adapters	1400
Linear guide rails, stepper motors	4500
Electronics and power supplies	400
Cables and drag chains	500
Sensors, lasers, limit switches	100
PC	1000
Software: Zerene Stacker Professional	265
Software: Agisoft Metashape Pro Educational	503
Total in Euros	9368

### Control electronics and software

Motion is controlled by an Arduino Mega microcontroller driving stepper motor drivers. The acquisition workflow is orchestrated by Python v. 3.10 communicating with the Arduino via serial (PySerial 2024, https://github.com/pyserial/pyserial). Image capture and download are performed using gphoto2 (gPhoto2 2024, www.gphoto2.org) invoked via the command line. The software logs axis positions, commands movements to each viewpoint in a predefined sequence, acquires focus stacks at each viewpoint, and stores all captured frames in a session folder for downstream processing. A relay-controlled power reset for the camera dummy battery is available to recover from occasional camera lockups during unattended acquisition (documented in the technical package).

### Viewpoint sampling strategy

Camera viewpoints were specified as discrete poses in a CSV “sequence” file (Orbitoscope repository: sequence.csv). A base camera-orbit path (X, Y, Z, A, B) was first programmed interactively using the live camera view to keep the specimen within frame and to ensure sufficient overlap between adjacent views for photogrammetric alignment. This base path was then replicated with three constant Z-offsets to increase viewpoint density and reduce occlusions, resulting in a total of 488 predefined viewpoints (sequence indices 0–487). The same 488-viewpoint sequence was used for all specimens in this study. Automated, geometry-aware viewpoint planning was not used here and is a target for future development (see Discussion).

### Sharpness metric and focus stacking acquisition

At each viewpoint, the system acquires a focus stack by stepping the C-axis and estimating image sharpness with an edge-based score computed from OpenCV Canny edge detection (Bradski 2000). Canny hysteresis thresholds were set to 0.0005 (low) and 0.002 (high) in normalized intensity units. A frame is treated as “in focus” when the resulting edge-based score exceeds a focus criterion determined empirically from pilot stacks acquired using the same optics, illumination, and exposure settings; this criterion was chosen to separate true specimen edges from persistent background noise. Stack acquisition continues through the in-focus region and terminates after ~10 consecutive frames fall below the focus criterion, which provides a conservative margin beyond the focal region while avoiding unnecessary captures. The exact implementation and parameter values are documented in the open-source code (Orbitoscope repository; sequenceD.py, execute_sequence_no_lasers()). Focus stacks were combined into a single EDOF image per viewpoint using Zerene Stacker (Professional, version T2024-11-18-1210); alternative open-source stacking tools are discussed in the Discussion.

### Photogrammetric reconstruction and scaling

**Figure 3.**
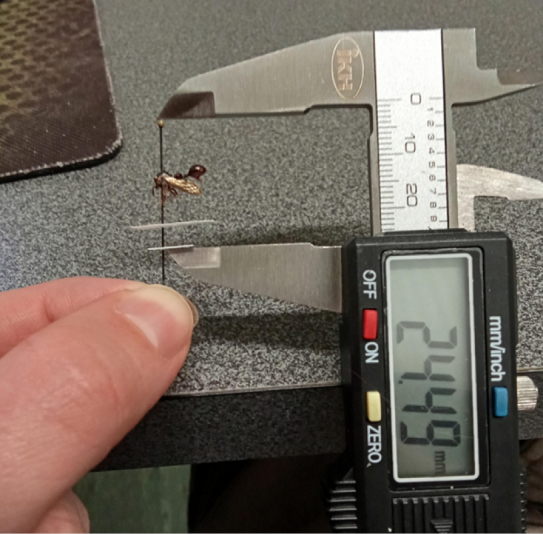
Adding scale to the 3D model. The distance of two known points is measured with a digital caliper.

EDOF images were reconstructed into textured 3D models using Agisoft Metashape (Agisoft 2024, v. 2.1.2). The reconstruction workflow uses standard structure-from-motion and multi-view stereo functions; a Metashape Standard license is sufficient for the core steps (we used an Educational license available to us). Models were scaled by marking two landmarks on the specimen mount corresponding to a known physical distance measured with digital calipers and applying this scale constraint during reconstruction (Fig. 3). Measurements can be performed within Metashape or after export in Blender (v. 4.2.3).

### Specimens digitized

Six pinned insect specimens were digitized, specimens were kindly provided by Villu Soon and Oleg Borodin. Links to interactive models (PlutoF and Sketchfab) are provided in Table 2.

**Table 2.** Specimens digitized.

PlutoF Identifier	Scientific name	Sketchfab link
TUZ247224	Coniocleonus nigrosuturatus	https://skfb.ly/psVEs
TUZ058737	Buprestidae Leach	https://skfb.ly/pt8Oq
TUZ047614	Membracidae Rafinesque	https://skfb.ly/pt8NJ
TUZ367101	Halictidae Thomson	https://skfb.ly/ptqYY
DUBD-0004680	Pyrgonota bifoliatus Westwood	https://skfb.ly/ptrPD
DUBD-0004685	Centrochares horrificus Westwood	https://skfb.ly/ptXSB

### Quantitative validation: 1D measurement accuracy

To provide a basic quantitative check of dimensional accuracy, three linear distances on one specimen (TUZ247224) were measured independently using a Leica stereomicroscope imaging system (Leica MC170 HD; Leica Application Suite LAS Core v. 4.12.0) and compared to the corresponding distances measured on the scaled 3D model in Blender (Fig. 7). Absolute error was computed as (3D – reference), and relative error as |3D – reference| / reference × 100%, using microscope measurements as the reference.

### Experimental collision-prevention module (not used in this study)

A prototype laser-based collision-prevention module was developed to interrupt motion if the camera approaches the specimen. This module was not used in the acquisition workflow for the results presented here; details are therefore provided in Suppl. material 1.

## Results

**Figure 4.**
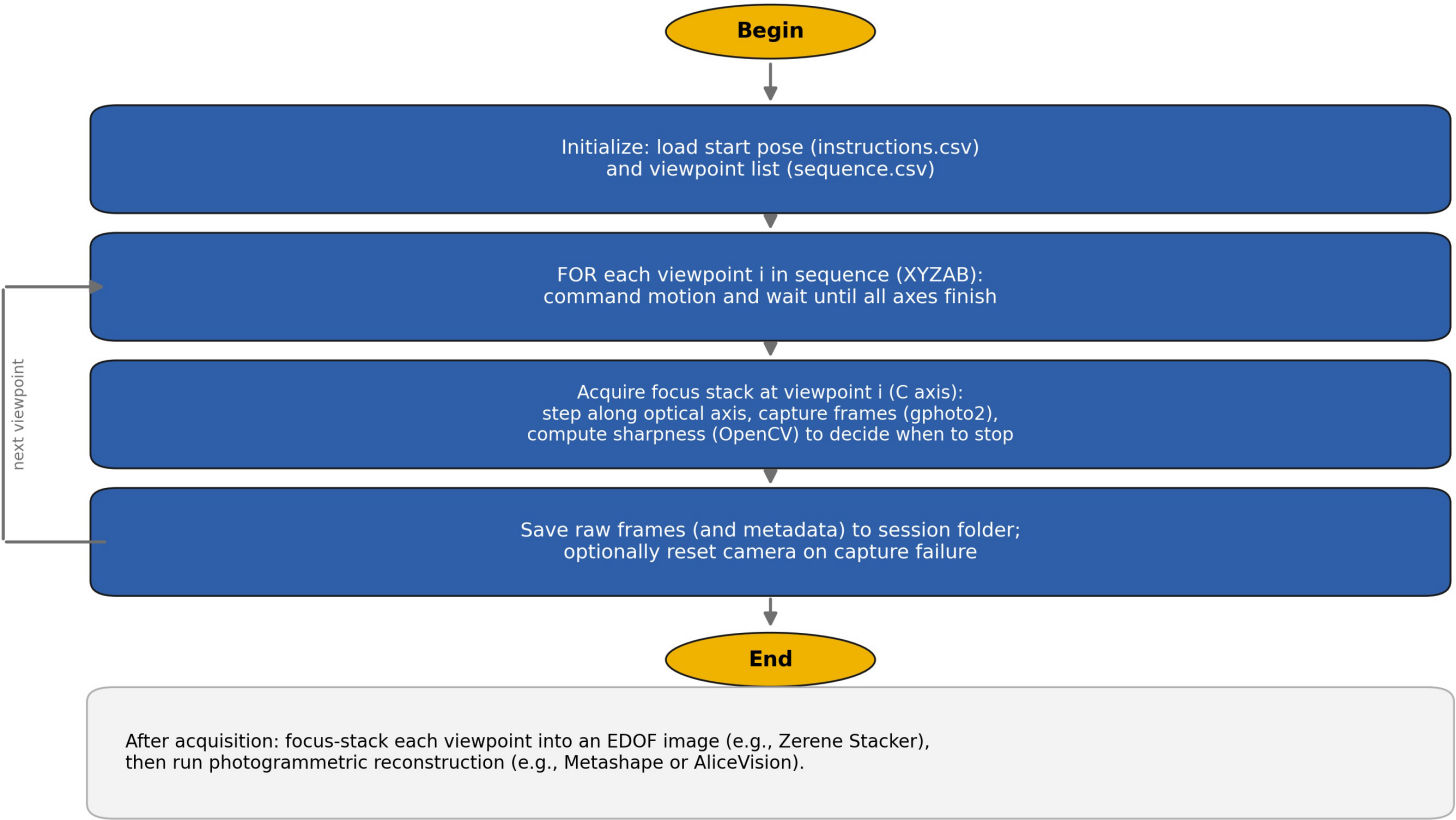
Imaging workflow flowchart.

Six pinned insect specimens were digitized using the Orbitoscope workflow (Fig. 4). For each specimen, the robot visited 488 predefined camera viewpoints (sequence indices 0–487). At each viewpoint, the C-axis was stepped to acquire a focus stack, producing one extended depth-of-field (EDOF) image per viewpoint after stacking. Individual stacks typically contained ~20–70 raw frames depending on specimen depth and the focus-search termination criterion.

**Figure 5.**
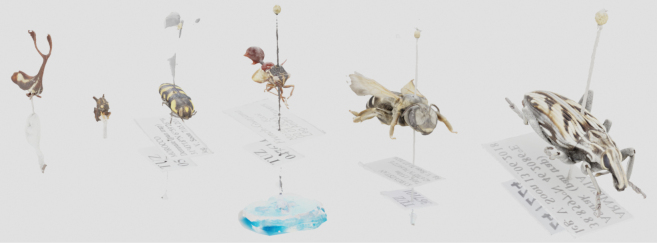
Six scanned specimens, scaled, textured mesh. In order from left: DUBD-0004680, DUBD-0004685, TUZ058737, TUZ047614, TUZ367101, TUZ247224.

**Figure 6.**
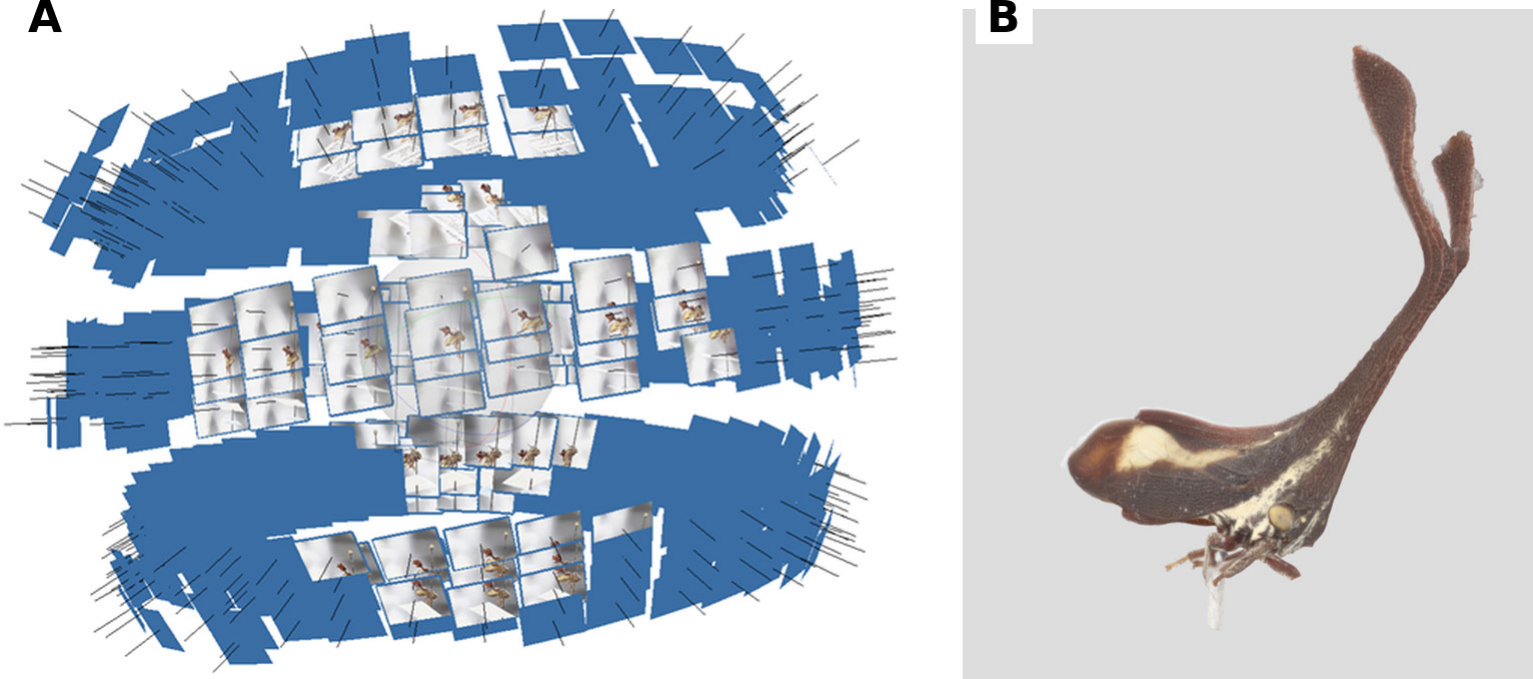
**A**. Example camera network / viewpoints from Structure-from-Motion alignment in Agisoft Metashape for one specimen, illustrating three elevation layers around the specimen; **B**. Resulting scaled, textured 3D model exported after reconstruction.

Across specimens, the total number of raw frames ranged from 12,000 to 26,000, and total acquisition times ranged from 42 to 67 hours per specimen (Table 3). Stacked EDOF images were successfully reconstructed into scaled, textured 3D models using photogrammetry (Figs 5, 6a, b). The resulting models were uploaded to online repositories (Sketchfab and PlutoF) to facilitate dissemination and reuse.

**Table 3.** Imaging times.

Specimen	Images taken	Imaging time (h)
TUZ367101	26000	67
TUZ247224	20500	55
TUZ058737	15500	42
DUBD-0004680	19500	49
DUBD-0004685	12000	57
TUZ047614	21000	56

### Quantitative 1D measurement validation

**Figure 7.**
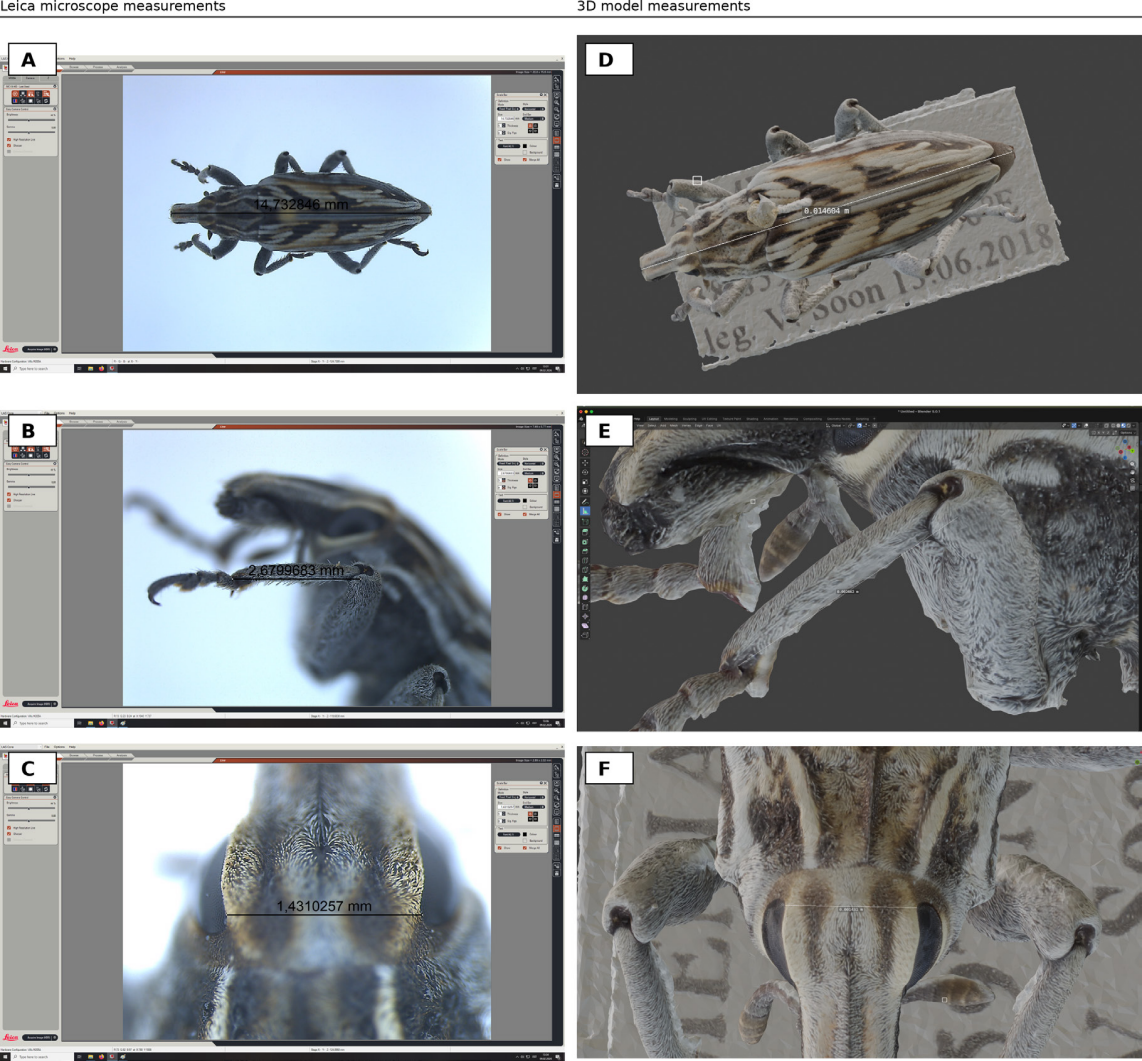
Validation of 1D measurements from scaled 3D models against microscope reference measurements. **A–C**. Linear distances measured on a pinned specimen using a Leica stereomicroscope imaging system: **A**. Total body length; **B**. Leg segment length; **C**. Inter-ocular distance. **D–F**. Corresponding distances measured on the scaled textured 3D model in Blender. Across the three distances, relative differences between 3D and microscope measurements ranged from 0.0018% to 0.875% (mean absolute percent error 0.516%).

For specimen TUZ247224, relative differences between 3D-model measurements and calibrated microscope reference measurements ranged from 0.0018% to 0.875%, with a mean absolute percent error of 0.516% (MAE 49.0 µm, RMSE 75.1 µm) (Fig. 7). These results indicate sub-percent agreement for representative anatomical distances when models are properly scaled.

## Discussion

This study presents a six-axis macro-imaging workflow in which the specimen remains stationary while the camera orbits around it using three translational axes (X–Y–Z) and two rotational axes (A–B), with a dedicated stacking axis (C) for automated focus stacking. Building on earlier robotic photogrammetry systems for insect digitization (Nguyen et al. 2014; Galantucci et al. 2016; Erolin et al. 2017; Ströbel et al. 2018; Cobb et al. 2019; Brecko and Mathys 2020; Doan and Nguyen 2024), our design shifts viewpoint variation from specimen motion (rotation/tilt) to camera motion. This reduces the need to handle and reorient fragile mounts and supports imaging of heavy, elongated, or potentially deformable objects. Because the camera can be positioned freely in 3D space, the orbit centre (aiming point) is not constrained to a single fixed rotation axis, allowing more flexible viewpoint selection and targeted high-detail imaging of specific regions when needed.

### Quantitative performance and measurement suitability

We provided a basic dimensional check by comparing measurements from a scaled 3D model to calibrated microscope measurements for specimen TUZ247224. The observed sub-percent deviations (MAPE 0.516%, maximum 0.875%) suggest that, under the imaging conditions used and with appropriate scaling, Orbitoscope-derived models can support practical morphometric measurements. This validation is limited in scope (three distances on one specimen) and should be expanded in future work to include additional specimens, additional landmarks, and ideally external reference standards (e.g., a stage micrometer or gauge object acquired within the same workflow).

### Practical limitations: size, setup effort, and throughput

The current system is physically large and heavy and requires dedicated space and robust support. Assembly and disassembly are time-consuming and typically require two people, particularly when handling the heavier gantry components. These practical constraints should be weighed against the flexibility gained from six-axis camera motion. Future iterations could reduce the footprint and improve portability by redesigning the frame and optimizing the distribution of moving mass.

### Viewpoint planning and calibration burden

In the current implementation, viewpoint sequences are defined manually using the live camera stream to keep the specimen centred and appropriately framed. Creating an initial sequence for a new specimen size class required approximately ~3 hours; additional layers (e.g., Z-offsets) can then be generated by applying constant offsets to the recorded coordinates. While effective, this manual step limits throughput and partially offsets the flexibility provided by six-axis motion. A major priority for future development is to streamline setup and usage by introducing automated, geometry-aware viewpoint planning based on a user-defined specimen envelope (e.g., bounding box/cylinder) or coarse geometry estimation (e.g., quick preview scan). A lightweight GUI that visualizes the planned camera path, guides alignment, monitors acquisition, and provides basic quality-control feedback would substantially improve usability and better realize the intended flexibility of the six-axis design.

More advanced sensing could support this: for example, a depth camera or other ranging sensor could be used to estimate specimen extent and clearance margins, detect potential collisions, and automatically generate camera poses that satisfy overlap and working-distance constraints—especially for larger or non-standard objects.

### Image sharpness, vibration, and smaller specimens

At higher magnification, residual vibration after motion can reduce sharpness and degrade focus stacking and photogrammetric reconstruction. In practice, this is mitigated through conservative motion speeds and a settling delay prior to capture. Imaging substantially smaller specimens (or using microscope objectives) will increase sensitivity to vibration; mechanical stiffening, improved damping/isolators under the frame, and motion profiles optimized for rapid settling are likely to improve performance in high-magnification regimes. Cross-polarized illumination and more controlled lighting placement may also help stabilize appearance and reduce specular artefacts that can interfere with feature matching.

### Reconstruction limitations and surface properties

As with other photogrammetric pipelines, reconstruction quality depends on image sharpness, exposure consistency, overlap, and viewpoint diversity, as well as specimen appearance. Specular, translucent, or low-texture surfaces remain challenging due to view-dependent appearance changes and reduced feature stability. Thin structures such as hairs and antennae are difficult to reconstruct reliably, especially where self-occlusion occurs. Emerging view-dependent reconstruction and rendering approaches (e.g., Gaussian splatting) may provide complementary representations for strongly view-dependent appearance (Kerbl et al. 2023), although they do not directly replace mesh-based workflows in all downstream applications (e.g., topology-dependent measurements or 3D printing).

### Software ecosystem, openness, and future improvements

Robot control and image acquisition are fully open-source. In this study, we used proprietary tools for focus stacking (Zerene Stacker) and photogrammetric reconstruction (Agisoft Metashape) due to speed and workflow maturity; however, open alternatives exist and may be substituted depending on project constraints. For example, AliceVision (3.3.0) provides a complete open photogrammetry pipeline (Griwodz et al. 2021), and open focus-stacking tools include Focus-Stack (Aimonen 2024, version 1.5) and shinestacker (Lista 2026, version 1.14.3). Additional workflow improvements include capturing RAW images to improve tonal flexibility and more robust colour processing, and further development of the laser-based collision-prevention concept before it is used in routine digitization.

## Conclusion

The Orbitoscope is an open-source six-axis macro-imaging robot that acquires photogrammetric image sets by orbiting a camera around a stationary specimen while performing automated per-view focus stacking via a dedicated stacking axis. Using this workflow, we digitized six pinned insect specimens and produced scaled, textured 3D models suitable for digital preservation, online dissemination, and measurement. A basic quantitative validation on specimen TUZ247224 showed sub-percent agreement between model-derived and calibrated microscope measurements (MAPE 0.516%, max 0.875%), supporting the use of Orbitoscope models for practical morphometrics when properly scaled. Future work will focus on reducing manual calibration effort through automated, geometry-aware viewpoint planning and a streamlined user interface, improving robustness for high-magnification imaging through vibration mitigation, and expanding quantitative benchmarking across more specimens and landmark types. All control software and documentation are released openly, enabling replication and adaptation. All code and documentation are available via GitHub and the archived technical package (Zenodo doi: https://doi.org/10.5281/zenodo.18563286).

## Additional information

### Use of AI

Grammarly was used to proofread this manuscript.

### Author ORCIDs

Toivo Aukusti Ylinampa https://orcid.org/0009-0007-4081-8092

Urmas Kõljalg https://orcid.org/0000-0002-5171-1668

### Data availability

All code is available in GitHub and Zenodo: https://github.com/ToivoYlinampa/Orbitoscope; https://doi.org/10.5281/zenodo.18563286. Example models generated with the described 3D scanner and processing pipeline are available at Sketchfab: https://sketchfab.com/toivo.ylinampa and at PlutoF.

## Supplementary materials

Supplementary material 1 Orbitoscope Data type docx https://binary.pensoft.net/file/1595063This dataset is made available under the Open Database License (http://opendatacommons.org/licenses/odbl/1.0/). The Open Database License (ODbL) is a license agreement intended to allow users to freely share, modify, and use this Dataset while maintaining this same freedom for others, provided that the original source and author(s) are credited. Toivo Aukusti Ylinampa, Urmas Kõljalg

Supplementary material 2 “Entodig 3D aka “The Höskä” Data type mp4 Explanation note **video 1**. “Entodig 3D aka “The Höskä” sneak peek timelapse.” Overview of the robot. A pinned insect is mounted on a specimen holder, which is then placed in front of the camera. The orbitoscope makes a “ring” around the object while capturing EDOF images. The image series is stacked together in Zerene Stacker, and the 3D model is calculated in Agisoft Metashape. Note: the video shows the C-axis camera movement with video stream. This is only for representation purposes; actual image series are captured using still images. https://youtu.be/JqL3NUszWyU?si=klpozpiDhZvKlT4o. https://binary.pensoft.net/file/1595064This dataset is made available under the Open Database License (http://opendatacommons.org/licenses/odbl/1.0/). The Open Database License (ODbL) is a license agreement intended to allow users to freely share, modify, and use this Dataset while maintaining this same freedom for others, provided that the original source and author(s) are credited. Toivo Aukusti Ylinampa, Urmas Kõljalg

Supplementary material 3 A six-axis macro-imaging robot, camera movement sneak peek Data type mp4 Explanation note **video 2**. A six-axis macro-imaging robot, camera movement sneak peek. Second overview of the robot. In addition to the movement shown in the first video, this video shows the tilting and height movements, performed by the B and Z axes. https://youtu.be/6bY5UTAF6HY?si=zzHIt45gIwzF37L9. https://binary.pensoft.net/file/1595065This dataset is made available under the Open Database License (http://opendatacommons.org/licenses/odbl/1.0/). The Open Database License (ODbL) is a license agreement intended to allow users to freely share, modify, and use this Dataset while maintaining this same freedom for others, provided that the original source and author(s) are credited. Toivo Aukusti Ylinampa, Urmas Kõljalg

Supplementary material 4 3D measurement from scaled insect models, using Blender Data type mp4 Explanation note **video 3**. 3D measurement from scaled insect models, using Blender. 3D measurements. The scaled models can be downloaded and opened in Blender or other 3D software. https://youtu.be/Q-LF-GNhxM8. https://binary.pensoft.net/file/1595066This dataset is made available under the Open Database License (http://opendatacommons.org/licenses/odbl/1.0/). The Open Database License (ODbL) is a license agreement intended to allow users to freely share, modify, and use this Dataset while maintaining this same freedom for others, provided that the original source and author(s) are credited. Toivo Aukusti Ylinampa, Urmas Kõljalg
