# Supplementary material for: The Orbitoscope, a six-axis macro-imaging robot for photogrammetric 3D-digitization of insects and other small specimens
Source: Zookeys. 2026 Apr 14;1277:137–55. doi: 10.3897/zookeys.1277.177740 (PMC13100664; doi:10.3897/zookeys.1277.177740)
Supplement: Supplementary material 1 — Orbitoscope [file zookeys-1277-137_article-177740__-s001.docx]

The point of this workflow is to move the camera around the object, hereby referred to as “orbiting” the object. The system performs this by moving the camera in six motorized movement axes in an automated fashion. Collected images are stacked and used for 3D reconstruction with separate software.

We used an Ubuntu pc config for robot control and imaging: i7 8-core 5 GHz, 16 GB DDR4, NVIDIA GTX1080. Following data analysis, we performed focus stacking and photogrammetric 3D reconstruction with an Apple M1 Max, 32 GB. We have uploaded all Python and Arduino code to GitHub, and it is available under the MIT license: https://github.com/ToivoYlinampa/Orbitoscope

Axis nomenclature

In previously published three-axis systems, the specimen panning and tilting (X and Y axes) are transformed into camera panning and tilting, hereby re-labeled as A and B axes. The traditional Z axis, which is the camera movement front and back, is hereby re-labeled as the C axis. This renaming of axes is because the X, Y, and Z axes are reserved for 3-dimensional camera movement in the presented system, as these axes represent the nomenclature of 3D-software coordination. The X-axis moves the camera apparatus front and back, and the Y-axis moves it left and right. The Z-axis lifts the camera apparatus up and down.

Hardware structure

We constructed the apparatus from off-the-shelf parts, but we needed machine tools. The system is quite heavy (about 80 kilograms), requiring good support and space for the camera apparatus to move around the object. Apparatus dimensions are 1.5 x 1.5 x 3 (width, length, height) meters. Assembling and disassembling take at least two persons, especially when lifting a heavy Z-axis onto the Y-axis. The hardware costs under ten thousand euros. The price will be higher if educational licenses are not available. The overall structure in Image 1 and Image 2.


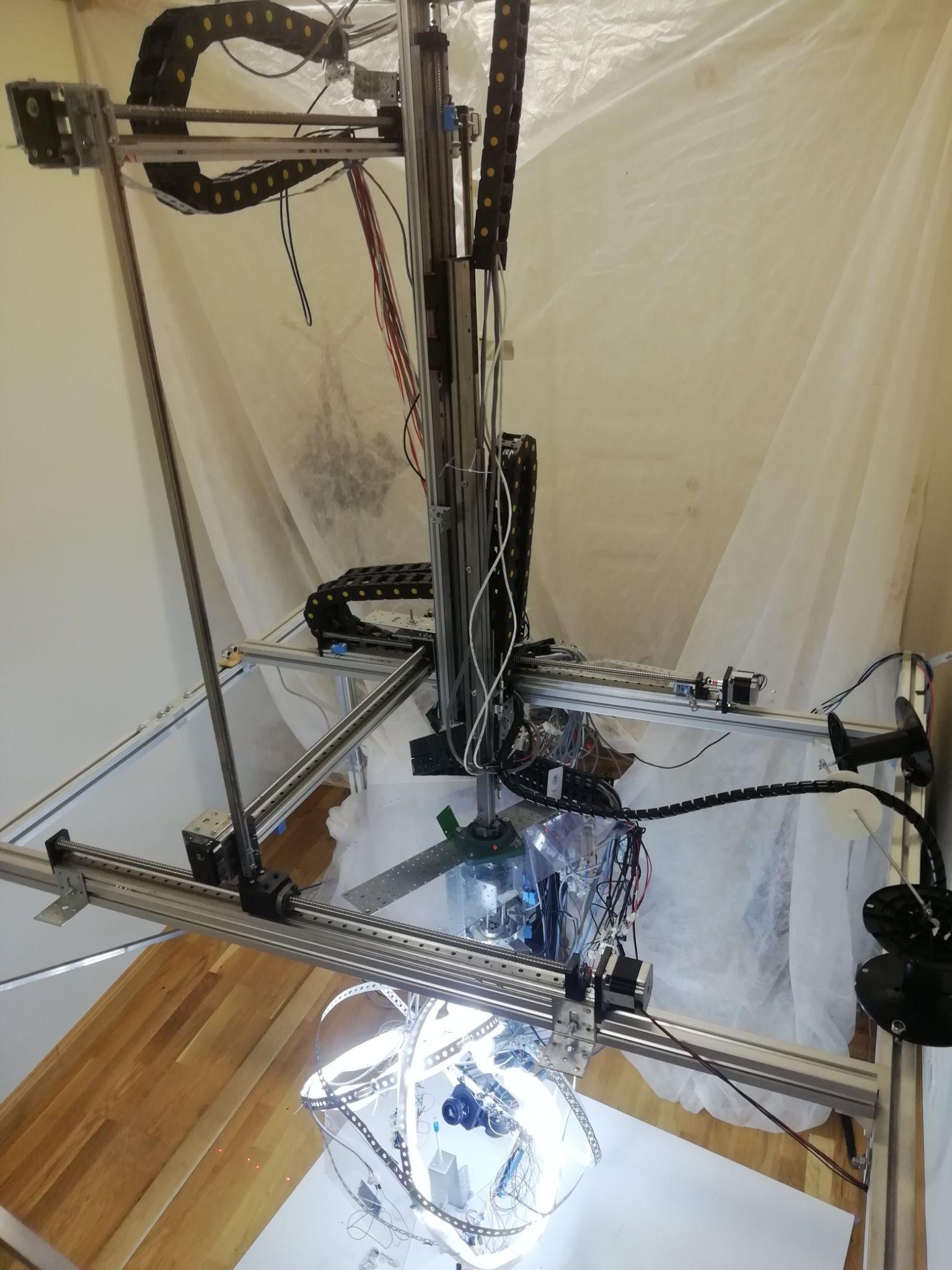


Image 1: Overall structure of the robot


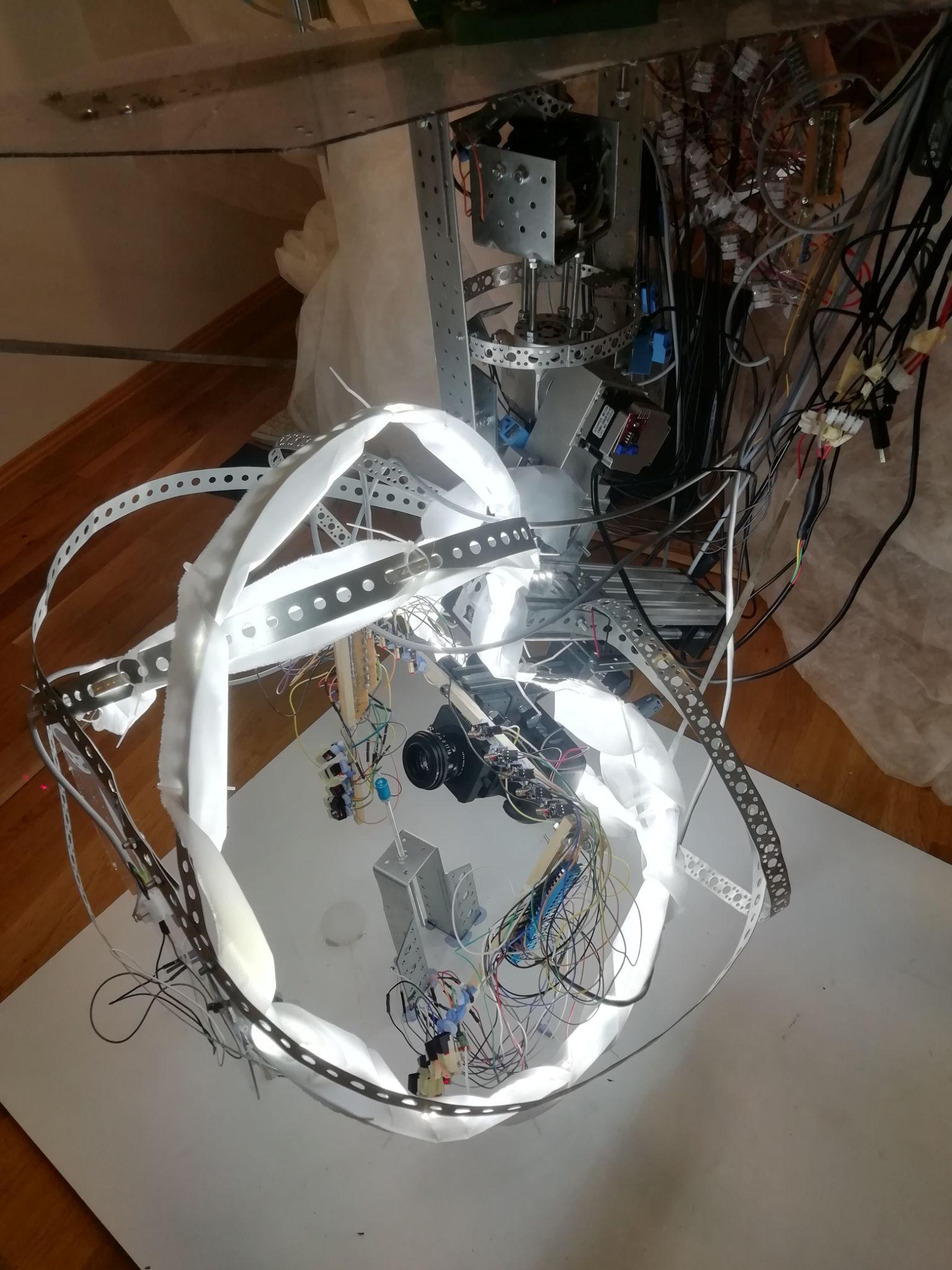


Image 2: Camera rotating and tilting apparatus

1.1 The base

Under the frame, there are wood blanks that will provide the base of the imaging apparatus. In the center of these wooden blankets is a wooden square holding the specimen mounting rod. The square has marks for the specimen holding rod and specimen mount-aiming device, which we made with Blu-Tack.

1.2 Specimen mounting rod

The specimen mounting rod has a metal base, about 25cm width and length, 40cm height, metal screw (m6), on top of which there is a hollow cylinder with two tightening screws. With a bit of Blu-Tack, the pinned specimen pin is placed into the cylinder so that the specimen center is precisely 17mm in height (or other height, depending on the calibration). The center of the Specimen should be at this height, but it can also be a large specimen so that the downmost specimen surface can be lower than that.

1.3 Specimen mount-aiming device

The specimen mount-aiming device has a metal base, about 40cm in height, and two pieces on top, making a 90-degree angle to the sides. Two lasers (KY-008) are attached to the metal with small m3 screws and blu-tack. These lasers aim at the center point, where the user should plant the Specimen. Lasers have three pins: 5V, GND, and signal. We wired these to an Arduino UNO with a simple code to keep these two lasers on whenever the power is provided. Arduino has a power supply that provides the power.

1.4 Frame

On top of the base, wood blanks lie on the aluminum frame. The aluminum frame type is 45x45, which is quite thick, to support the heavy weight and to reduce vibration. We fastened the frame to the wood blank base with bolts and screws. The frame comprises eight aluminum frames, each about 1 to 1,5 meters long. The height of the frame is 120cm, the length is 150cm, and the width is 150cm.

1.5 X-axis

On the frame lies the X-axis. It consists of a Linear guide rail with a corkscrew slide. This robust structure eliminates vibration and can withstand heavy loads. It is also more durable than a chain structure, which loosens its tight grip as the chain elongates over time. The movement is actuated by stepper motors (2-phase, 1.8° step size, NEMA 23). These motors are very accurate (200 steps in one turn). For more durable movement and to reduce shakiness, two pieces of linear guide rails and motors were placed on the right and left sides of the X-axis. Both motors are connected to the same stepper motor driver and thus receive identical movement pulses. This stepper motor driver has extra amperage (3A) because more energy is needed to sustain the movement of two motors instead of one. The wires to these motors go along the frame towards the electronics board, which will be explained later. For calibration, limit switches (aka end-stoppers) are placed at the beginning and end of the linear guide rail. As the movement of linear guide rails is actuated, when the linear guide slide approaches the beginning of the linear guide rail, the limit switch (SN-04) will detect the slide inductively and note the microcontroller with a signal. The same can be done to detect the maximum position on the opposing side. Going to the “zero-position” is a crucial step when starting the scanning to ensure that the calibration is done right and the scanning is always done precisely. The loosening of the calibration is affected by stepper motor kick-back phenomena or when executing movement that is too fast with too heavy loads.

SN-04 has four wires: 5V, GND, and signal wire. 5V and GND are shared with other Arduino peripherals, but the signal wire goes to the Arduino digital pin in the electronics board. The purpose of the X-axis is to move the whole imaging apparatus in the X-axis, meaning front and back. In our setup, the movement area was 80cm, which was actuated in 120 thousand steps, Nema 23, 2-phase. SFU1605, 800mm. Next to the side of the X-axis, which is nearer to the electronics table, is a cable drag chain that holds all the wires needed in the mentioned axes. The start of the cable drag chain is fixed to the aluminum profile frame over the electronics board, where all the cables go. The hind end of the cable drag chain is fixed to the X-axis linear guide rail, so it moves with the X-axis slide.

1.6 Y-axis

The Y-axis is attached to the X-axis slider, moving with it on the left and right sides. The Y-axis also has two sets of linear guide rails and stepper motors, which are vertical. The first is directly on top of the X-axis slider, and the second is about one meter higher on top of metal bars, whose base is attached to the X-axis sliders. The Y-axis moves the imaging apparatus from left to right in a similar fashion to the X-axis. Another cable drag chain is attached to this stage to withhold cables.

1.7 Z-axis

Attached to the two Y-axis linear guide rail sliders is the Z-axis. (FSL40E80010C7 800mm) This stage lifts the imaging apparatus up and down. There are double rails attached to just one slider, 80cm long. Only one motor (NEMA 25), more potent than the previously mentioned axes, supports the heavy lifting against gravity. This motor should be fastened when the power is cut; when the power is lost, there is no holding torque, and the imaging apparatus will slowly fall against the ground, which might damage the specimen and the imaging apparatus itself. A holding clip can be placed between the stepper motor and the base of the corkscrew, which will safely keep the Z-axis slider in the air when the power is cut off. From the Z-axis, an elongated steel piece is bolted; it will go over the slider to elongate the movement range of the Z-axis. A cable drag chain will cover all the cables.

1.8 A-axis

The A-axis has a stepper harmonic drive and a high torque gearing system (NEMA 25, 1:50, TRNC11-A50-C50) attached to the end of the Z-axis steel block. It rotates the imaging apparatus. Harmonic drive has a 1:50 gearing that will enable more precise movement and high torque. The motor block of the A-axis rotates around and moves the axes mentioned later. The spinning phase of the motor is attached to the Z-axis slider block, so it does not rotate in relation to the Z-axis slider. The cables are enclosed in a double-bending cable drag chain, whose start end is fixed to the Z-axis slider, and the end of the double-bending cable drag chain is fixed to the side of the rotating plastic base. The plastic base is fixed to the A-axis motor block, so it rotates in relation to the Z-axis slider block. This ensures the full rotation of the cable drag chain. The double-bending cable drag chain is a regular drag chain, but a piece of the drag chain is removed to enable the bending in both ways. To get the “zero-position,” there is a metal screw attached to the rotating plastic base. The limit switch is bolted to a swivel, which will be opened and put in front of the metal screw when calibrating. After the calibration, the swivel is brought back to prevent collisions. A ball-bearing ring is placed between the rotating plastic base and the Z-axis slider to improve the structure and reduce vibration. An additional piece of plastic hangs from the rotating plastic base, in which additional electronics are attached. The entire round is 40 thousand steps in the current setup.

1.9 B-axis

Similar to the A-axis, another harmonic drive is attached (NEMA 25, 1:50). This motor tilts the imaging apparatus. The rotating side is connected to the A-axis base, and the base of the B-axis is attached to the C-axis. The limit switch is placed at the starting end of the axis movement range. At zero position, the B-axis points about 30 degrees upwards. At the maximum, at the 14 thousand steps, the B-axis points about 30 degrees downwards. In between these, the B-axis is leveled.

1.10 C-axis

The C-axis is attached to the bottom of the B-axis. It has a stepper motor that pushes and pulls the camera stage front and back to gain full depth of field (SFU1605,100mm). It has limit switches for calibration. On the C-axis moving slider, a PB-6 bellows is attached. The bellows allow the movement of the lens in relation to the camera, effectively zooming the images. An El-Nikkor 50mm 2.8 enlargement lens is attached. This lens has excellent enlargement for an affordable price. Attached to the lens is a small lens hood to reduce glowing from lights. A camera (Canon 70D) is attached to the other end of the bellows. The camera has a dummy battery, which provides power to the camera via a cable. A USB cable is attached to the camera to transfer images and observe the video stream. Into the C-axis, a laser-X collision prevention system is attached (Image 3, Laser-X fork).


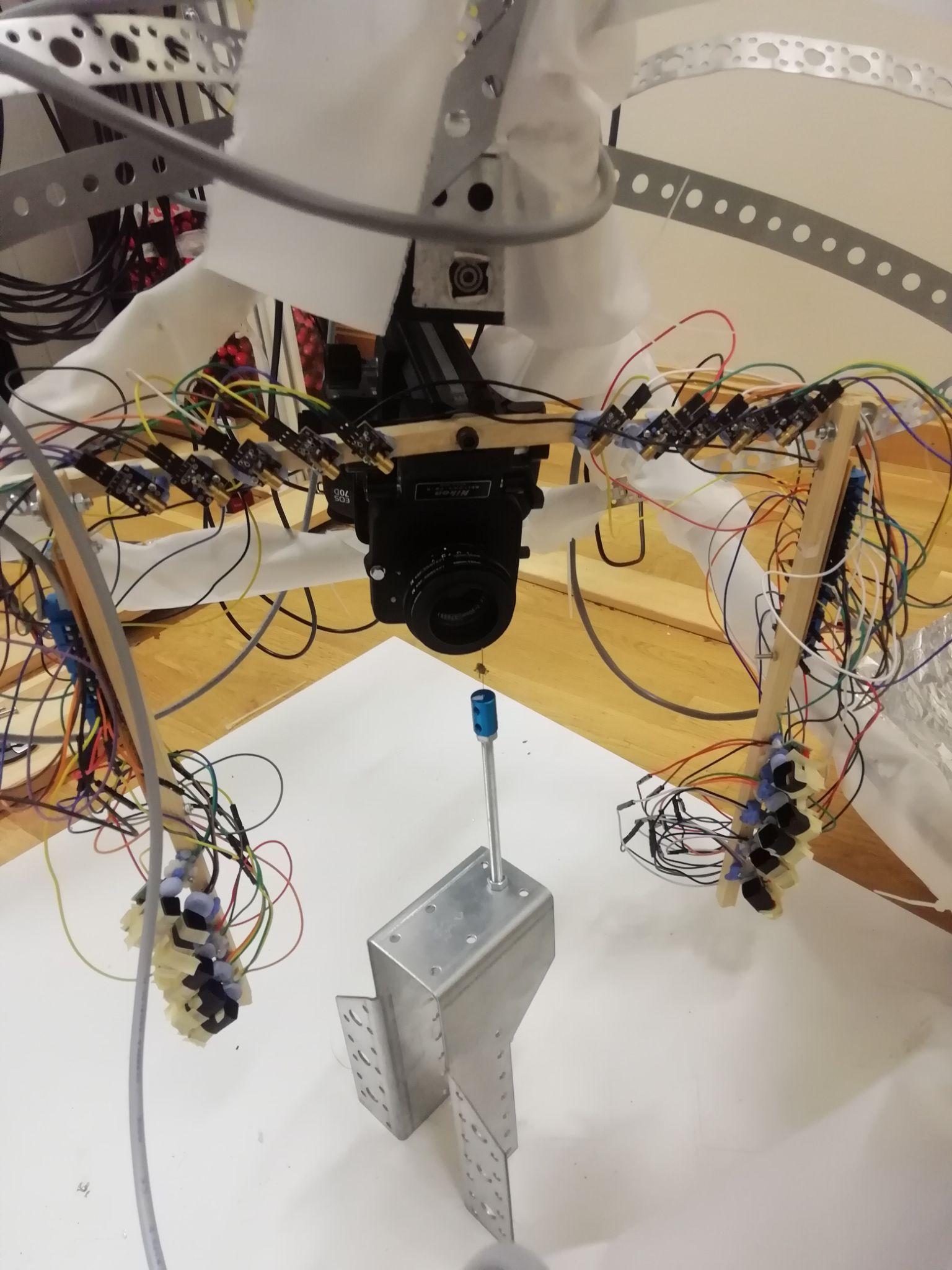


Image 3: Laser-X fork

The Laser-X is experimental and not currently used in imaging. It has ten lasers providing beams in front of the lens, going to the detectors on the opposite side. If the camera comes too close to the specimen, the laser beam to the detector is interrupted, and the movement can be stopped. The lasers and sensors are attached to a “fork,” which holds the cables. The detector has three pins: 5V, GND, and the signal. The laser is detected with a photodiode, which lets the signal go through if enough light is applied. To prevent false detections, a cube is built around the photodiode. The front has a 1cm x 1cm 650nm band-pass filter, which lets only the red light, mainly from the lasers, through. The other walls of the cube are 1cm x 1cm mirrors to ensure that the red light that comes through the band-pass filter keeps bouncing in the cube enough to get a higher chance of hitting the photodiode. In the C-axis, a metal fixer is attached (Image 4, light sphere). It makes a ring around the specimen imaging area, and an LED light strip is attached to it to provide even lighting. The LED lights are 6500K white light. To soften the light, a white textile (softbox textile) is placed to cover the LED light strip. This light softening prevents hard shadows and evens the light. Also attached to this fastener ring is a led matrix, which could be used to get silhouettes of the specimen if needed. Finally, the fastener rings can be wrapped with aluminum foil to keep the light in the imaging dome.


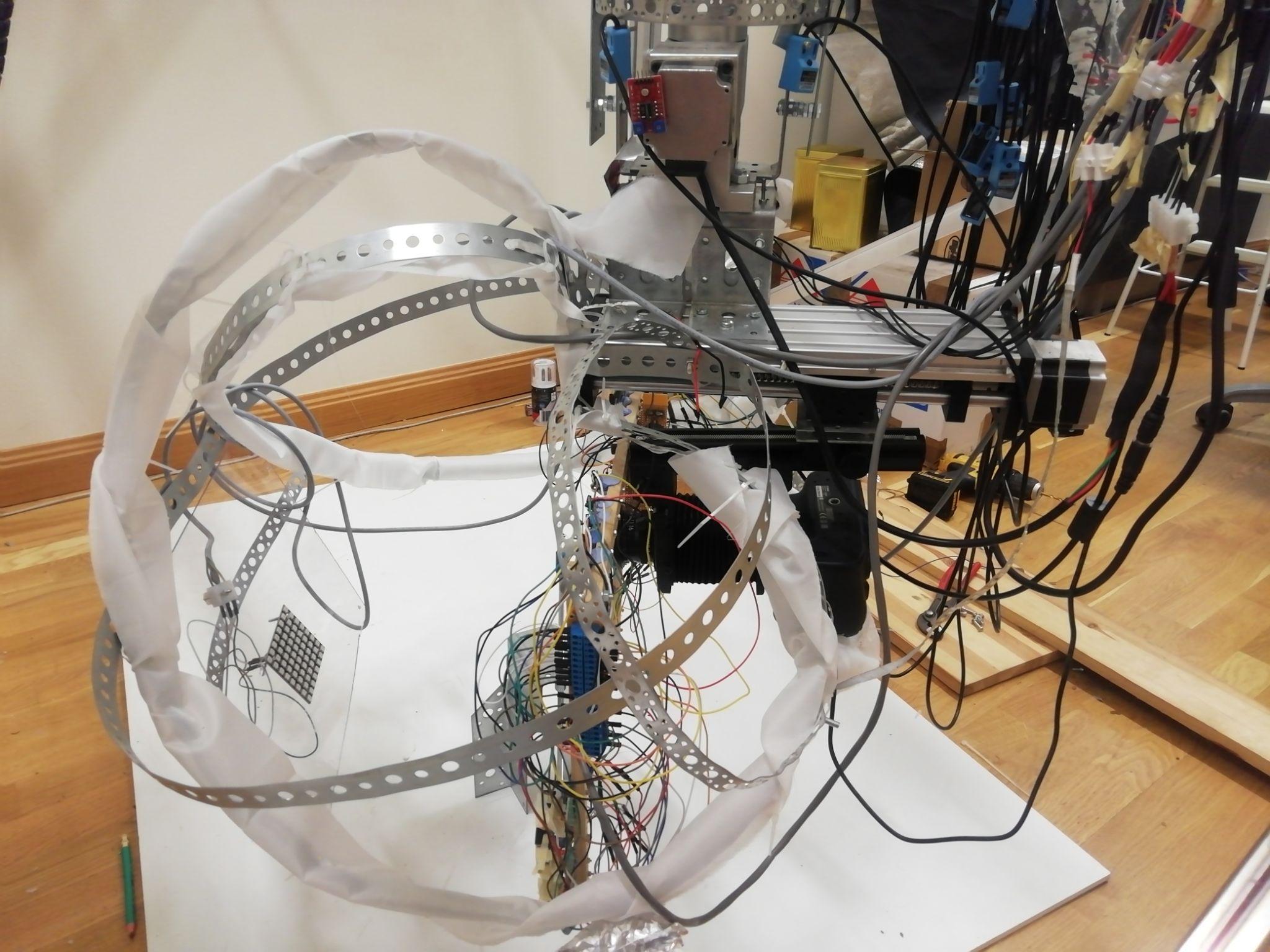


Image 4: Light sphere

1.11 Electronics board

All the cables go to the electronics board, except the USB cable from the camera, which goes to the PC. An Arduino Mega microcontroller is connected to the PC, from which the Python program sends movement commands to the Arduino. Arduino software keeps track of each of the six motor axis positions and orchestrates those movements. Arduino also powers and orchestrates information from all sensors, including all the axes' minimum and maximum position limit switches (except the A-axis, which has just one limit switch), 10 lasers, and detectors from the laser-X fork. Arduino is also connected to 6 stepper motor drivers, which are in turn connected to all six sets of stepper motors. Axes X and Y have two motors and thus slightly increased amperage to provide enough power. Stepper motor drivers are TB6600, configured to 1,5A and 2A. 24V power supply provides power to the stepper motor drivers. A separate 12V power supply provides power to the LED lights. Arduino Mega has its power supply also. The camera dummy battery is connected to a relay switch, which restarts the camera if needed.

Cabling: connect Stepper Motor Driver to Arduino following the Arduino’s code, for example on line 4:

AccelStepper stepperX(1, 4, 5); // DIR:3, STEP: 2

Means that Stepper Driver’s Dir wire should be connected to Arduino’s pin 5, and the STEP wire should be connected to Arduino’s pin 4.

2 Software

The imaging process utilizes open-source software. However, after the images have been acquired, the following stacking and photogrammetry processes use proprietary software, which is faster and produces better results. The PC runs a GNU/Linux operating system (Ubuntu 22.04 LTS).

2.1 Image capture

For digital camera control, image, and potential video capture, Gphoto2 ([www.gphoto2.org](http://www.gphoto2.org)), an open-source utility for Linux, is used. It captures the data and downloads it into the PC via various means (e.g., USB cable) using command line functions. It can also be used to change image capturing settings, like ISO values and speed shutter times. However, the lens aperture must be adjusted manually in this setting. Gphoto2 is used to capture JPEG images, and it is also used to stream video in the calibration phase. In this study, we captured images with the following settings: ISO 400, f/8, 1/100. The main Python program calls Gphoto2 via CLI (Command Line Interface) in the subprocess.

2.2 Python main program

The Imaging process is orchestrated by the main Python program (python 3.10). It coordinates the camera movement, launches camera operations, stores images in Ubuntu pc, and analyzes images. The photo stacking and photogrammetry are done manually after imaging. The main program communicates with the Arduino Mega microcontroller via USB cable with a pyserial (<https://github.com/pyserial/pyserial>) library. Python program opens instructions.csv file, which includes the current positions for all six axes. Python program also opens the sequence.csv file, which contains all the coordinates for each camera position. When imaging, the primary Python program creates a session folder where all the images are saved. Python main programs analyze each image with Opencv and calculate its sharpness values to check if the image is in focus. The Python main program controls the camera via a USB cable with the Gphoto2 library. Camera settings can be changed with this library, but it is usually used to launch the image-taking process and transfer the new image into the computer. Numpy (Oliphant 2006) and OpenCV (Bradski 2000) libraries were used.

2.3 Arduino program

Arduino Mega is an open-source electronics platform (Arduino.cc). The scripts are written with Arduino IDE, and it has a serial communication system that can communicate with Python via the Pyserial library. Accelstepper library was utilized for motor actuation (Airspayce 2024). Arduino keeps track of each axis position, receives movement orders from the Python program, and actuates those movement orders. Arduino also checks the signals from various end stoppers.

Arduino commands via serial monitor are structured in the following way: First comes the axis, in capital if we are doing movement to relative position (X, Y, Z, A, B or C), and lower case if we are doing movement to absolute position (x, y, z, a, b or c). Should the movement be counter-clockwise, meaning negative direction, the - (minus) symbol comes right after the axis character. Then comes the amount of steps. And finally, after a space comes the speed in seconds. For example, “X-50 40” moves the x-axis in relation to the current position 50 steps in a negative direction with a speed of 40 steps in a second. As a second example, “y60 30” moves the y-axis into an absolute value of 60 steps, with a speed of 30 steps in a second. Other commands include homing or going in the negative direction until the corresponding limit switch is triggered. This is then labeled as zero-position. These “min” position commands are “H” for the x-axis, “I” for the y-axis, “J” for the z-axis, “K” for the a-axis, “M” for the b-axis, and “N” for the c-axis. Arduino also has commands to “set” and “get” values of each axis. With the command “w,” the Arduino gets or tells the current position of the x-axis and prints it into the serial communicator. With “W” and some value, the x-axis value can be set to Arduino. For example, “W400” tells the Arduino that the x-axis value is 400. Similar commands are for other axes: “e” for the y-axis, “r” for the z-axis, “t” for the a-axis, “o” for the b-axis, and “p” for the c-axis.

2.4 Imaging process

The Python main program starts and contacts Arduino. Python checks the current camera position from instructions.csv and passes that information to the Arduino. Python creates a new folder for acquired images. Python checks the first index positions from sequence.csv and passes those coordinates to Arduino, which starts the movement to that XYZAB position. Python waits until Arduino confirms that the movement is done. Then Python starts the C-stack process. Assuming the robot calibration has been done correctly, the specimen should be in the middle of the frame. Now, the camera has to be moved front and back to gain the full depth of field, "Extended Depth Of Field, EDOF" -image. Python sends the image-capturing command to the camera via Gphoto2 to get the "Initial image." If the camera is not responding, some other processes may be claiming the camera and the camera has to be restarted. In this situation, the Python restarts the camera by sending commands to the Arduino to turn the camera off and on again by cutting the power down to the camera dummy battery by a relay switch. After the camera is restarted, the Python program stops other processes that are using the camera (kill -9).

After the initial image has been taken and transferred successfully to the folder, Opencv detects the image sharpness value using Canny edge detection. If the initial image is in focus, meaning that the Canny value was over the threshold, it means that the image was in focus, and the camera has to be moved backward as long as there is something in focus. This is done by sending C-axis movement commands to the Arduino, which moves the camera. After a "big step backwards" a new image is taken, its sharpness is analysed, and the camera keeps moving backwards until there is nothing in focus anymore. Then, the camera starts moving forward, collecting images throughout the sharp area until nothing is in focus anymore. Thus, the whole specimen has been imaged, and the camera can be moved to its original position. Later, these images can be stacked together for an EDOF image. If the initial image is not in focus, then the sharp area is searched first by moving the camera backward a couple of times and then forward a couple of times. If sharpness was not found, or the image series was captured successfully, the camera is moved to the next index position. When all camera position indices have been passed through, the process is finished.

Camera XYZAB position sequence was with three elevation's orbit, and each position's value was multiplied by three by giving an offset for the Z-position values.

Calibrating the Robot

As the camera moves freely in six dimensions, it must be calibrated to always point towards the specimen. Calibration is done by manually collecting XYZAB-camera position values. This is done by moving the camera robot via Arduino IDE serial communicator and opening the camera's video stream either by gphoto2 stream or simply by opening the camera's screen. Then, the desired sequence positions are collected by moving the camera via Arduino commands.

Use E.py python program, and with command “stream” the Gphoto/Python starts video stream. Then, using Arduino serial monitor, move the camera. If E.py/stream stops working, restart the stream by killing the program (ctrl-c). Usually the operating system grabs the camera, so before the streaming is possible, use the E.py “freeCamera” command, it uses camera related process’es kill –9, to free the camera. After the video streaming is done, use the command “stopStream” to stop streaming.

When the single camera position was correct, the positional values were asked from Arduino, and values were stored in a table sheet (example in supplementary material: table.txt). Then, the values were converted to CSV with script tableToCsv.py, thus providing table.csv. The same script can also give extra movement position in the z-axis in the script example.

Before the imaging, the starting position should be inserted into the "instructions.csv" file. The robot should be moved manually to the starting position using Arduino serial communication. Finally, the imaging can be initiated by running "sequenceDC.py", waiting for it to initialize communication with the Arduino, and then typing command "sequence no lasers". Note that all the Python libraries have to be installed beforehand. Also, the Arduino serial communicator should be closed before imaging so the serial communication is free for the Python program.

References

Airspayce (2024) AccelStepper Arduino library. Available from: https://www.airspayce.com/mikem/arduino/AccelStepper/ (accessed on 15 October 2024)

Aimonen P (2024) *focus-stack* (software). Available from: [https://github.com/PetteriAimonen/focus-stack](https://github.com/PetteriAimonen/focus-stack?utm_source=chatgpt.com) (accessed on 15 October 2024). [github](https://github.com/PetteriAimonen/focus-stack?utm_source=chatgpt.com)

Bradski G (2000) The OpenCV library. *Dr. Dobb’s Journal of Software Tools* 120: 122–125.

gPhoto2 (2024) gPhoto2 — Digital camera software. Available from: [https://www.gphoto2.org](https://www.gphoto2.org/) [accessed 15 October 2024]

Oliphant TE (2006) *A guide to NumPy.* Trelgol Publishing, USA. <https://numpy.org/doc/stable/>

Orbitoscope (2025) <https://github.com/ToivoYlinampa/Orbitoscope> [accessed on 4 February 2025]

PySerial (2024) <https://github.com/pyserial/pyserial> [accessed on 15 October 2024]

Ubuntu (22.04 LTS). <https://ubuntu.com/desktop> [accessed on 15 October 2024].

Zerene Stacker (2024) [www.zerenesystems.com](http://www.zerenesystems.com/) [accessed on 15 October 2024]
